# Increased circulating microRNA-122 is a biomarker for discrimination and risk stratification in patients defined by sepsis-3 criteria

**DOI:** 10.1371/journal.pone.0197637

**Published:** 2018-05-21

**Authors:** Tim Rahmel, Simon T. Schäfer, Ulrich H. Frey, Michael Adamzik, Jürgen Peters

**Affiliations:** 1 Klinik für Anästhesiologie, Intensivmedizin und Schmerztherapie, Universitätsklinikum Knappschaftskrankenhaus Bochum, Bochum, Germany; 2 Klinik für Anästhesiologie und Intensivmedizin, Universität Duisburg-Essen and Universitätsklinikum Essen, Essen, Germany; National Yang-Ming University, TAIWAN

## Abstract

**Background:**

Sepsis is now operationally defined as life-threatening organ dysfunction caused by an infection, identified by an acute change in SOFA-Score of at least two points, including clinical chemistry such as creatinine or bilirubin concentrations. However, little knowledge exists about organ-specific microRNAs as potentially new biomarkers. Accordingly, we tested the hypotheses that micro-RNA-122, the foremost liver-related micro-RNA (miR), 1) discriminates between sepsis and infection, 2) is an early predictor for mortality, and 3) improves the prognostic value of the SOFA-score.

**Methods:**

We analyzed 108 patients with sepsis (infection + increase SOFA-Score ≥2) within the first 24h of ICU admission and as controls 20 patients with infections without sepsis (infection + SOFA-Score ≤1). Total circulating miR was isolated from serum and relative miR-122 expression was measured (using spiked-in cel-miR-54) and associated with 30-day survival.

**Results:**

30-day survival of the sepsis patients was 63%. miR-122 expression was 40-fold higher in non-survivors (p = 0.001) and increased almost 6-fold in survivors (p = 0.013) compared to controls. miR-122 serum-expression discriminated both between sepsis vs. infection (AUC 0.760, sensitivity 58.3%, specificity 95%) and survivors vs. non-survivors (AUC 0.728, sensitivity 42.5%, specificity 94%). Multivariate Cox-regression analysis revealed miR-122 (HR 4.3; 95%-CI 2.0–8.9, p<0.001) as independent prognostic factor for 30-day mortality. Furthermore, the predictive value for 30-day mortality of the SOFA-Score (AUC 0.668) was improved by adding miR-122 (AUC 0.743; net reclassification improvement 0.37, p<0.001; integrated discrimination improvement 0.07, p = 0.007).

**Conclusions:**

Increased miR-122 serum concentration supports the discrimination between infection and sepsis, is an early and independent risk factor for 30-day mortality, and improves the prognostic value of the SOFA-Score, suggesting a potential role for miR-122 in sepsis-related prediction models.

## Introduction

Sepsis is the primary cause of death from infection and one of the leading causes of intensive care unit (ICU) mortality, and prognosis has not improved markedly over the last years [1, 2]. Furthermore, survivors of sepsis often have significant physical, psychological, and cognitive disabilities causing a great burden for health care systems and societies [3].

Nevertheless, the understanding of sepsis and its pathobiology has improved, also evoking a new definition of sepsis [4]. Sepsis is now defined as life-threatening organ dysfunction, identified by an acute increase in the SOFA-Score by at least two points, likely occurring as the result of a dysregulated host response to an infection. The SOFA-score includes clinical chemistry items such as serum creatinine or bilirubin concentration [4]. Since early diagnosis seems crucial for improving survival in septic patients, molecular tracers may facilitate an even earlier diagnosis and hence fast treatment initiation [5–8]. Specifically, only scarce knowledge exists about more recently discovered regulatory microRNA (miR) family, 21–24 nucleotide length RNAs regulating gene expression [9], that as biomarkers might contribute to better prediction of survival in sepsis.

Since circulating MicroRNA´s are protected from degradation by inclusion in extracellular microvesicles [10] or formation of protein-microRNA complexes with Argonaute 2 [11], nucleophosmin [12], or HDL [13] circulating miRs are remarkably stable and their expression can be quantified in body fluids including blood making them attractive as potential biomarkers [14, 15]. miRs are also critically involved in innate and adaptive immunity with bacterial infections directly targeting the tumor necrosis factor pathway [14, 16] and toll-like-receptor / NF-κB signaling pathways, highlighting an interaction of miRs with the immune response in sepsis [14].

Circulating miR-122 is described as a tissue-specific RNA that represents almost 70% of all liver miRs and is only minimally expressed in other tissues [17]. Influencing liver cell differentiation, proliferation, and apoptosis via several genes, miR-122 was shown to be a specific biomarker for hepatocyte damage and liver disease [18, 19]. Recently, alterations of miR-122 serum concentrations have been linked to acute liver dysfunction in inflammatory diseases and sepsis [20–22] and were suggested to be a useful biomarker for early mortality-prediction according to the older Sepsis-1 definition [23]. Increased miR-122 expression occurred earlier and its increase was more specific to predict an acute liver injury in rats compared to an increased aspartate aminotransferase (AST) and alanine aminotransferase (ALT) activity or serum-bilirubin concentration [24]. miR-122 might also be a useful diagnostic biomarker indicating early acute liver dysfunction in sepsis and, therefore, possibly improves the detection of a sepsis-defining organ dysfunction. However, results from experiments in rodents showing a remarkable difference of miR122-expression between controls and septic mice 24 hours after caecum ligation and puncture [22], could not be reproduced to the same extent in a human cohort with sepsis-1 patients [23]. In this context, absence of organ dysfunction according the sepsis-1 definition could explain the great interindividual variability and the reduced predictive value of miR-122 expression in human studies [23]. Since the sepsis-3 definition, in contrast to sepsis-1 definition, focuses on organ dysfunction, the role of miR-122 in the context of sepsis should be reconsidered.

Accordingly, we tested the hypotheses that micro-RNA-122, 1) is useful for discrimination between patients meeting sepsis-3 criteria and infection, 2) is an early predictor of mortality, and 3) improves the prognostic value of the SOFA-score.

## Materials and methods

This study was reviewed and approved by the Ethics Committee of the Medical Faculty of the University of Duisburg-Essen (#06–3078) and written informed consent was obtained from patients or their guardians. Patients admitted to our ICU of the University of Duisburg-Essen Medical School were considered eligible if they fulfilled the Sepsis-1 definition (infection + ≥2 SIRS-criteria) and Sepsis-3 definition (infection + increase ≥ 2 in SOFA-score), and did not suffer from chronic liver disease [4, 25]. All patients were followed up for 30-day survival as calculated from day 1 of the diagnosis of sepsis. Patients were treated using a multimodal concept that included analgosedation, fluid administration, and protective mechanical ventilation as well as hemodynamic, antibiotic, and diagnostic management, as described previously [26].

Blood, besides clinical and demographic data on study entry, was sampled with the first 24 h after above mentioned criteria were met. In total, 108 patients with sepsis (64 males (59%), 44 females (41%), mean age: 55.6 years ± 15.7) were included. The observation period was defined from admission to our ICU either to day 30 of hospital stay or death. Clinical characteristics of this cohort are presented in Table 1. Samples were than analyzed en bloc for miR-122 and compared to the clinical data.

**Table 1:** Baseline characteristic of survivors and non-survivors (30 days).

Variable	Survivors n = 68 (63%)	Non-survivors n = 40 (37%)	P-value
Age *yrs*. (range/± SD)	55.0 (24-82/±15.8)	56.6 (18-86/±15.8)	0.620
Male gender (%)	39 (57)	25 (63)	0.599
Body mass index (*kg/m*^*2*^)	27.3 (±5.8)	26.9 (± 5.9)	0.815
Medical history, no. (%)			0.927
- Cardiovascular disease	16 (24%)	9 (23%)	
- Gastrointestinal disease	20 (29%)	11 (27%)	
- Gastrointestinal cancer	4 (6%)	2 (5%)	
- Hematooncological disease	1 (1%)	3 (8%)	
- Lung disease	14 (21%)	9 (23%)	
- Skin and soft tissue infection	3 (4%)	1 (2%)	
- Trauma	2 (3%)	1 (2%)	
- Urogenital disease	5 (8%)	3 (8%)	
- Other	3 (4%)	1 (2%)	
Dialysis (%)	30 (44%)	26 (65%)	0.036
Mechanical ventilation (%)	47 (69%)	29 (73%)	0.710
Horowitz-Index (P_a_O_2_/FiO_2_), (mmHg)	286.5 [207–377]	324 [188–382]	0.807
C-reactive protein concentration (*mg/dl)*	11.7 [5.8–18.7]	10.6 [3,7–20.2]	0.566
Procalcitonin concentration (*ng/m*l)	3.1 [1.1–12.7]	2.5 [1.0–18.3]	0.752
Interleukin-6 concentration (*pg/ml*)	77.1 [22.1–146.5]	234.0 [53.5–774.3]	0.007
Leukocyte concentration (*10*^*9*^*/l*)	12.5 [9.1–18.1]	17.1 [10.6–22.3]	0.108
AST activity (*U/l)*	59 [25–119]	81 [37.5–524.5]	0.006
ALT activity (*U/l)*	40 [20–96]	49 [25–364.5]	0.061
Total bilirubin concentration (*mg/dl)*	1.2 [0.5–2.2]	1.9 [1.1–7.3]	0.001
LDH activity (*U/l*)	349.5 [252.5–473]	480 [217–1119]	0.141
INR	1.2 [1.1–1.4]	1.4 [1.1–1.7]	0.034
Platelet concentration *(/nl)*	128 [72–196]	102 [52–158.5]	0.016
SAPS II	45.5 (±17.4)	47.8 (±17.7)	0.519
SOFA-Score	10.2 (±3.7)	12.4 (±3.6)	0.002
Gram positive isolates only (%)	29 (43%)	14 (35%)	0.689
Gram negative isolates only (%)	23 (34%)	12 (30%)	
Mixed bacterial isolates (%)	6 (9%)	8 (20%)	
Viral isolates (%)	0 (-)	0 (-)	
Fungal isolates (%)	1 (1%)	1 (2%)	
Negative cultures (%)	9 (13%)	5 (13%)	

Data are presented as n (%); means (± SD), medians (25th, 75th percentile), AST: Aspartate aminotransferase ALT: Alanine aminotransferase, LDH: Lactate dehydrogenase, INR: International Normalized Ratio; SAPS II: Simplified Acute Physiology Score; SOFA: Sepsis-related Organ Failure Assessment score.

### Control patients with infection

20 adult patients (9 men, 11 women, mean age 52.3 years ± 17.2) treated in the ICU for infections (infection + ≥2 SIRS-criteria) suffering from pneumonia (n = 18) and/or urinary tract infection (n = 2) but without sepsis according to the Sepsis-3 definition (SOFA-score ≤ 1) served as controls. Blood was sampled within the first 24 h after ICU admission. Further characteristics of the control group with infection in comparison to sepsis patients are summarized in S1 Table.

### Blood sample collection, preparation, and storage

Blood samples were collected in Vacuette® Serum Tubes and processed within 30 minutes. Following centrifugation for 10 minutes at 2500 rpm serum samples were shock frozen in liquid nitrogen and stored at -80°C until analysis. Sample aliquots were thawed for miRNA analysis to avoid degradation from multiple freezing and thawing procedures.

### Serum micro-RNA extraction

miR-122 was isolated from 400 µl serum using the miRVana PARIS Kit (Ambion, Life Technologies Corporation, USA) according to the manufacturer´s instructions. Due to the lack of validated reference miRs for normalization, 25 fmol exogenous cel-miR-54 from C. elegans (Qiagen, Hilden, Germany) was spiked into samples immediately before miR isolation. This allows adjustment for differences in sample preparation efficacies. Total RNA was eluted in 100 µl of RNase-free water and stored at -80°C until further use.

### miR-122 expression measured by quantitative PCR (qPCR)

Relative quantification of miR-122 was carried out using the qRT-PCR miR Detection Kit (Ambion®, Life Technologies, USA) and hsa-miR-122 PCR Primer Sets for amplification of the miR-122. qRT-PCR was performed using the Applied Biosystems Step One Plus Real-Time PCR System taking advantage of the Taqman miR Assays for cel-miR-54, miR-122, and the Taqman Universal Master Mix II no UNG (all Applied Biosystems, Carlsbad, USA) in a final volume of 20 µl including 1 µl cDNA from the RT reaction as template. Relative expression of miR-122 with cel-miR-54 as control was expressed using the comparative 2^-ΔCT^ method. PCR conditions were as follows: incubation of the samples for 10 min at 95°C followed by application of 40 cycles of 15 seconds at 95°C and 1 minute at 60°C and all samples were run in duplicate.

### Statistical analyses

Continuous variables are presented as means ± standard deviation (SD) in case of normal distribution and as median and interquartile range (25^th^; 75^th^ percentile) in case of non-normally distributed variables. Continuous variables were compared using parametric ANOVA (including Bonferroni Holm post hoc testing) or non-parametric Kruskal-Wallis statistics (followed by post-hoc Dunn´s Test). Categorical variables were characterized by numbers with percentage and were compared using the Chi-square test. Correlation between miR-122 expression and other variables was analyzed using the Spearman correlation test, and values of p<0.05 were considered statistically significant.

Predictive validity of bilirubin and SOFA-Score as well as miR-122 expression regarding 30-day mortality were assessed with receiver operator characteristics (ROC) and corresponding results for area under the curve (AUC). In a second step, ROC-analysis regarding 30-day mortality and sepsis vs. controls were used to define miR-122 cut-off values with the Youden-Index to discriminate between survivors and non-survivors, as well as between infection and sepsis. Furthermore, 30-day survival was displayed using Kaplan-Meier plots with univariate log-rank test for trend. A multivariable Cox-regression analysis including the variables with p<0.05 from univariate demographic statistics was used to determine whether categorized miR-122 expression was independently associated with 30-day survival. To avoid overfitting, a restricted model was assessed afterwards including the variables with p<0.1 from the initial model. Hazard ratios (HR) and 95% confidence intervals (CI) were calculated from the Cox regression analysis to describe the effect of covariates on the hazard. Finally, dichotomized miR-122 was added to the SOFA-Score as additional variable and ROC-curves of SOFA-Score + miR-122 were generated to evaluate the prognostic utility. Reclassification analyses using net reclassification improvement (NRI) and integrated discrimination improvement (IDI) were used to assess the added value of miR-122 to the SOFA-score. All analyses were performed using SPSS (version 24, IBM, Chicago, IL, USA), R (version 3.4.0, R Foundation for Statistical Computing, Vienna, Austria) and for graphical presentations GraphPad Prism 7 (Graph-Pad, San Diego, CA, USA) was used.

## Results

Table 1 shows the baseline characteristics upon ICU admission of the 108 patients with sepsis. The observed 30-day survival was 63% and median duration of ICU stay was 28 days (14 days; 39 days). Patients upon admission demonstrated a SOFA-Score of 11.0 (± 3.8) with a significant difference between survivors (10.2 ± 3.7) and non-survivors (12.4 ± 3.6; p = 0.002).

**Fig 1:**
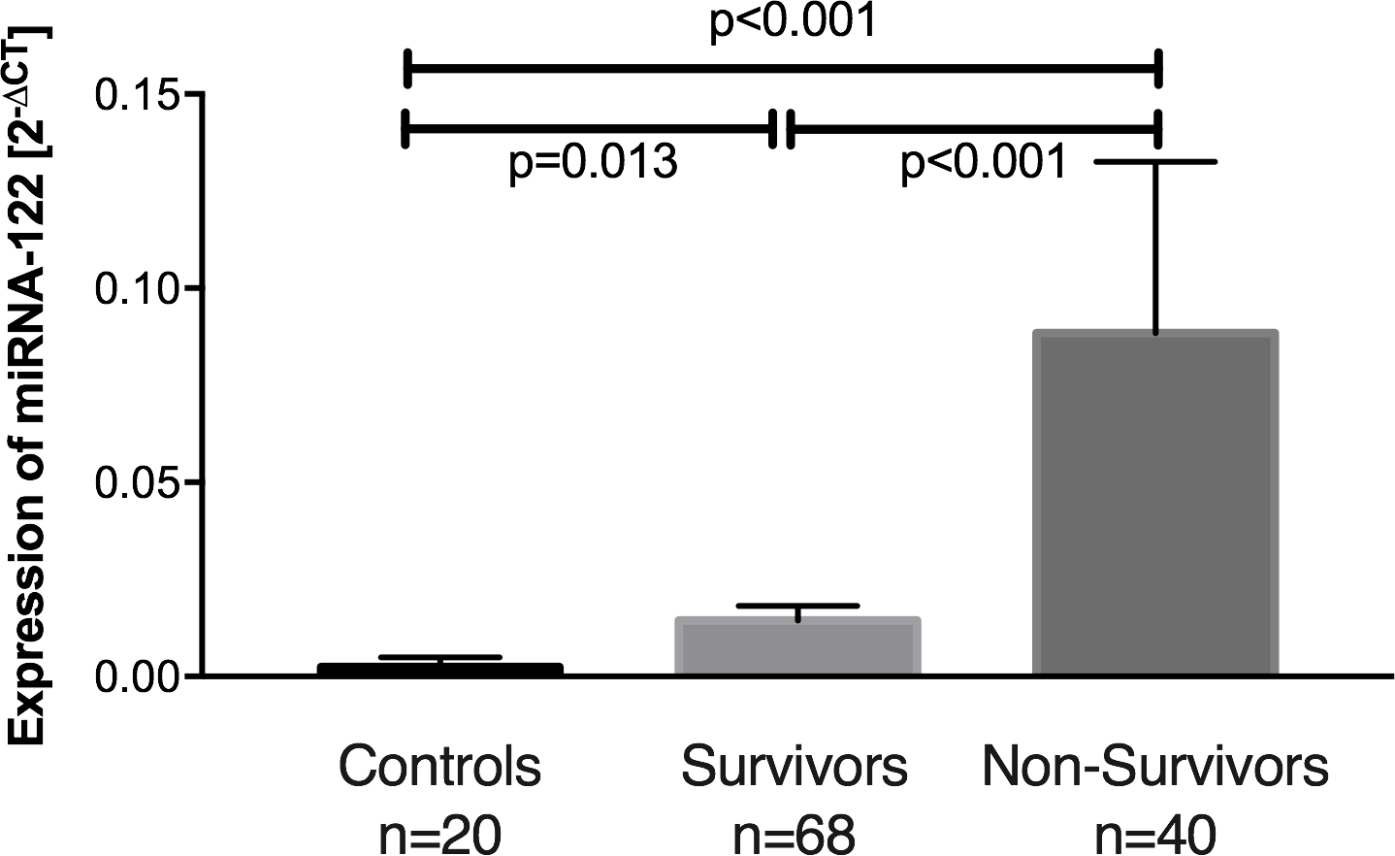
Relative miR-122 expression in control patients, sepsis survivors, and sepsis non-survivors. Means with standard error of the mean.

Expression of miR-122 on average was 18-fold increased in patients suffering from sepsis compared to controls (p<0.001), with a 40-fold increased miR-122 expression in sepsis non-survivors (p<0.001, Fig 1) and a 6-fold increase in survivors (p = 0.013, Fig 1). Thus, comparing survivors to non-survivors, we found an almost 6-fold greater miR-122 expression in non-survivors (p<0.001; Fig 1).

We next analyzed the impact of different variables on miR-122 expression by performing correlation analyses with clinical chemistry and hemodynamic values. In these analyses, miR-122 expression correlated with established markers of liver damage such as AST (p<0.001), ALT (p<0.001), and LDH (p = 0.009) as well as total bilirubin concentration (p = 0.002), and INR (p = 0.005). In contrast, we found no significant correlation of miR-122-expression with markers of cholestasis such as AP and GGT (Table 2).

**Table 2:** Correlation between relative miRNA-122 expression (ΔCT) with other laboratory markers and cardiovascular variables.

	*p value*	*Spearman´s correlation coefficient (r)*
***Liver damage***		
- AST activity	< 0.001	0.448
- ALT activity	< 0.001	0.502
- LDH activity	0.009	0.303
***Cholestasis & Liver function***		
- GGT activity	0.362	
- AP activity	0.501	
- INR	0.005	0.274
***SOFA-Score***	0.043	0.195
- Total bilirubin concentration	0.002	0.295
- Mean arterial pressure	0.041	-0.279
- Dosage of noradrenaline	0.002	0.297
- Dosage of epinephrine	0.156	
- Dosage of dobutamine	0.686	
- Platelet concentration	0.409	
- Serum-Creatinine concentration	0.147	
***Inflammation***		
- C-reactive protein concentration	0.114	
- Procalcitonin concentration	0.254	
- Interleukin-6 concentration	0.569	
- White blood cell concentration	0.397	
***Cardiovascular function & right heart burden***		
- Cardiac index	0.648	
- Stroke volume index	0.800	
- Mean pulmonary artery pressure	0.172	
- Central venous pressure	0.374	

AST: Aspartate aminotransferase, ALT: Alanine aminotransferase, LDH: Lactate dehydrogenase, GGT: Gamma-glutamyl transferase, AP: alkaline phosphatase, INR: International Normalized Ratio.

To analyze further potential triggers for increased miR-122 expression and its potential impact on the SOFA-Score we performed correlation analyses between miR-122 expression and the individual items of the SOFA-Score, markers of inflammation, global cardiovascular function, and right heart burden. In this context, miR-122 expression was not correlated with markers of inflammation or right heart burden (Table 2). However, we found a significant correlation between miR-122 and mean arterial pressure (p = 0.041) and noradrenaline dosage (p = 0.002).

The value of miR-122 expression to discriminate between infection and sepsis was evaluated using receiver-operating analysis (AUC: 0.760, p<0.001) and revealed a cutoff ratio of 0.004 (2^-ΔCT^) with a sensitivity of 58.3%, a specificity of 95.0%, a negative predictive value of 0.30, and a positive predictive value of 0.98.

**Fig 2:**
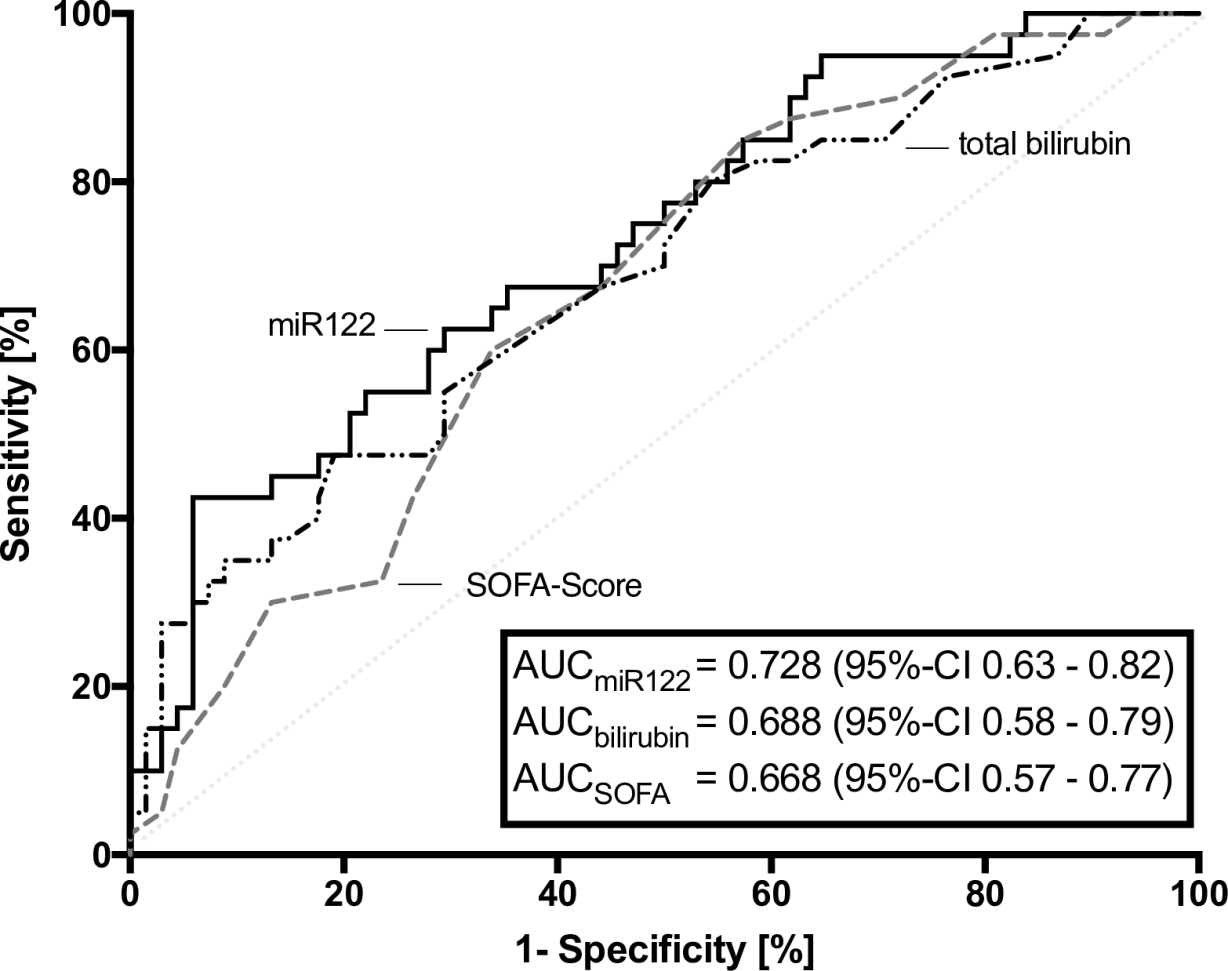
Receiver operating characteristics of miR-122 expression, total serum bilirubin concentration, and SOFA-score in relation to 30-day-mortality. Measurements on day 1 in patients with sepsis.

**Fig 3:**
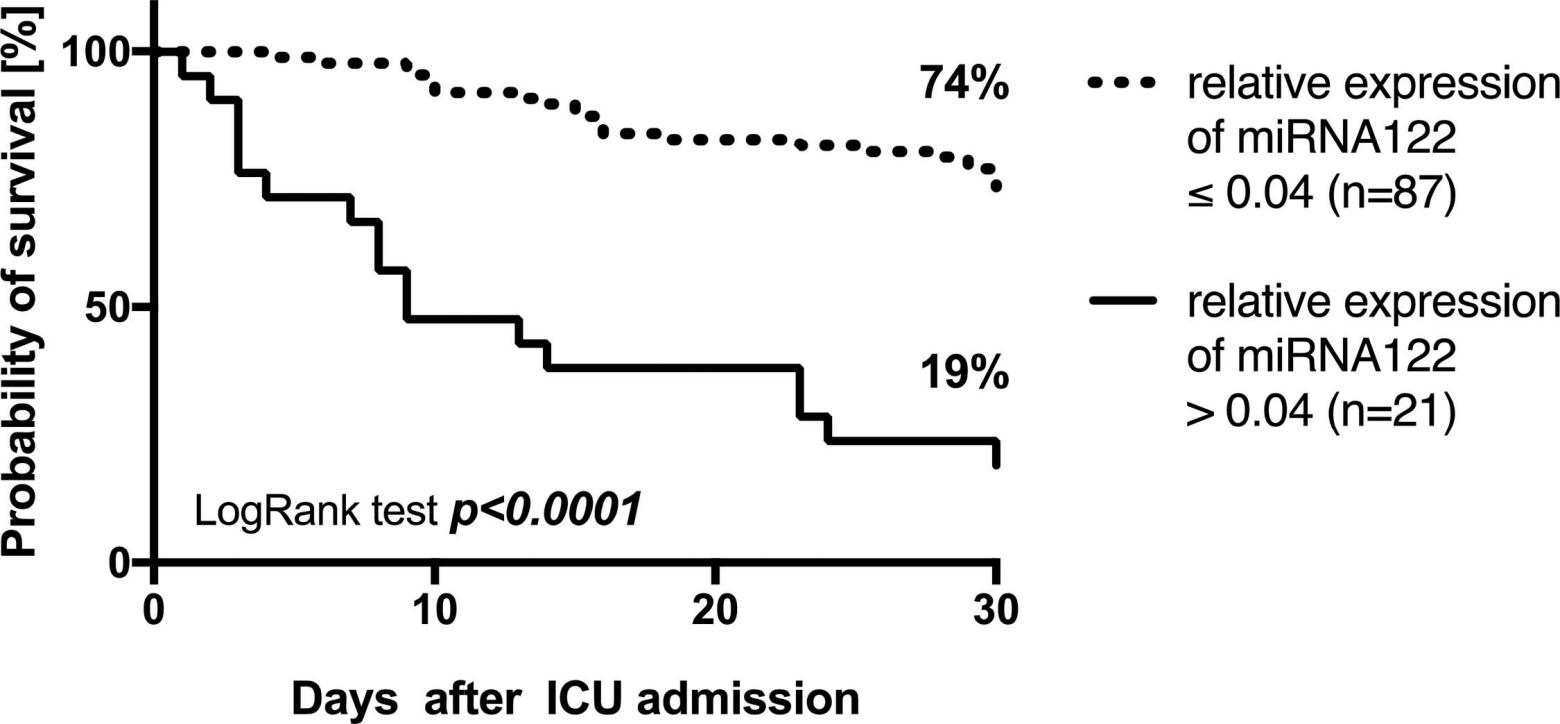
Kaplan-Meier estimates according thirty-day survival in patients with sepsis stratified for miR-122 expression.

The prognostic value of miR-122 expression to predict 30-day mortality was evaluated next and revealed a cutoff ratio of 0.04 as the value with the highest sum of sensitivity and specificity, to discriminate between sepsis survivors and non-survivors. miR-122 expression revealed (AUC: 0.728, p<0.001, Fig 2) a 2^-ΔCT^ of 0.04 resulted in a sensitivity of 42.5%, a specificity of 94.1%, a negative predictive value of 0.74, and a positive predictive value of 0.81. Using this cut-off, 30-day survival could be calculated as 74% for patients with a 2^-ΔCT^ ≤ 0.04 but only 19% for patients with a 2^-ΔCT^ > 0.04 (p<0.001, Fig 3).

To further compare the value of circulating miR-122 serum-levels as biomarker for early prediction of 30 day survival, receiver operating characteristic curves were generated for total bilirubin concentration and SOFA-score on day 1. Comparing mortality-prediction variables with miR-122 expression with AUCs as a measure of assay reliability were 0.728 for miR-122 (p<0.001), 0.688 for total bilirubin concentration (p = 0.001), and 0.668 for SOFA-score (p = 0.004; Fig 2). According to nonparametric testing by the DeLong test we found no significant p-values between the AUCs of miR-122 vs. bilirubin (p = 0.581) and miR-122 vs. SOFA-score (p = 0.411).

Multivariable Cox-Regression analysis referring miR-122 to dialysis, SOFA-Score, platelet count, total bilirubin concentration, AST and ALT activities, INR, PTT, and Interleukin-6 concentration as covariates exposed that patients with miR-122 expression above the cut-off of 0.04 had a hazard ratio of 3.9 in the initial model (95%-CI 1.6–9.1, p = 0.002) and a HR of 4.3 (95%-CI 2.1–8.9, p<0.001) in the restricted model and, therefore, showed significant impact on 30-day survival (Table 3).

**Table 3:** Cox regression analysis in patients with sepsis stratified for miR-122 expression.

(Co) variable	Multivariate					
	Initial			Restricted		
	p-value	HR	95%- CI	p-value	HR	95%- CI
miR-122 ≤ 0.04 [2^-ΔCT^]	-	1		-	1	
**miR-122 > 0.04 [2**^**-ΔCT**^**]**	**0.002**	**3.867**	**1.635–9.143**	**<0.001**	**4.300**	**2.080–8.888**
Dialysis [no]	-	1				
Dialysis [yes]	0.447	1.410	0.582–3.417			
SOFA score [per unit]	0.935	1.005	0.894–1.129			
Platelet concentration [10^9^/l]	0.967	1.000	0.996–1.004			
**Bilirubin concentration [mg/dl]**	**0.064**	**1.081**	**0.995–1.175**	**0.014**	**1.082**	**1.016–1.152**
AST activity [U/l]	0.154	1,000	0.999–1.001			
**INR**	**0.089**	**1.920**	**0.906–4.066**	**0.004**	**2.311**	**1.310–4.079**
Interleukin-6 concentration [pg/ml]	0.585	1.000	1.000–1.000			

HR: Hazard ratio point estimates, 95% CI, and p-values (two-sided) from Wald tests are reported, 2^**-**ΔCT^: relative expression level of miRNA 122, SOFA: Sepsis-related Organ Failure Assessment score, AST: Aspartate aminotransferase, PTT: Partial thromboplastin time.

**Fig 4:**
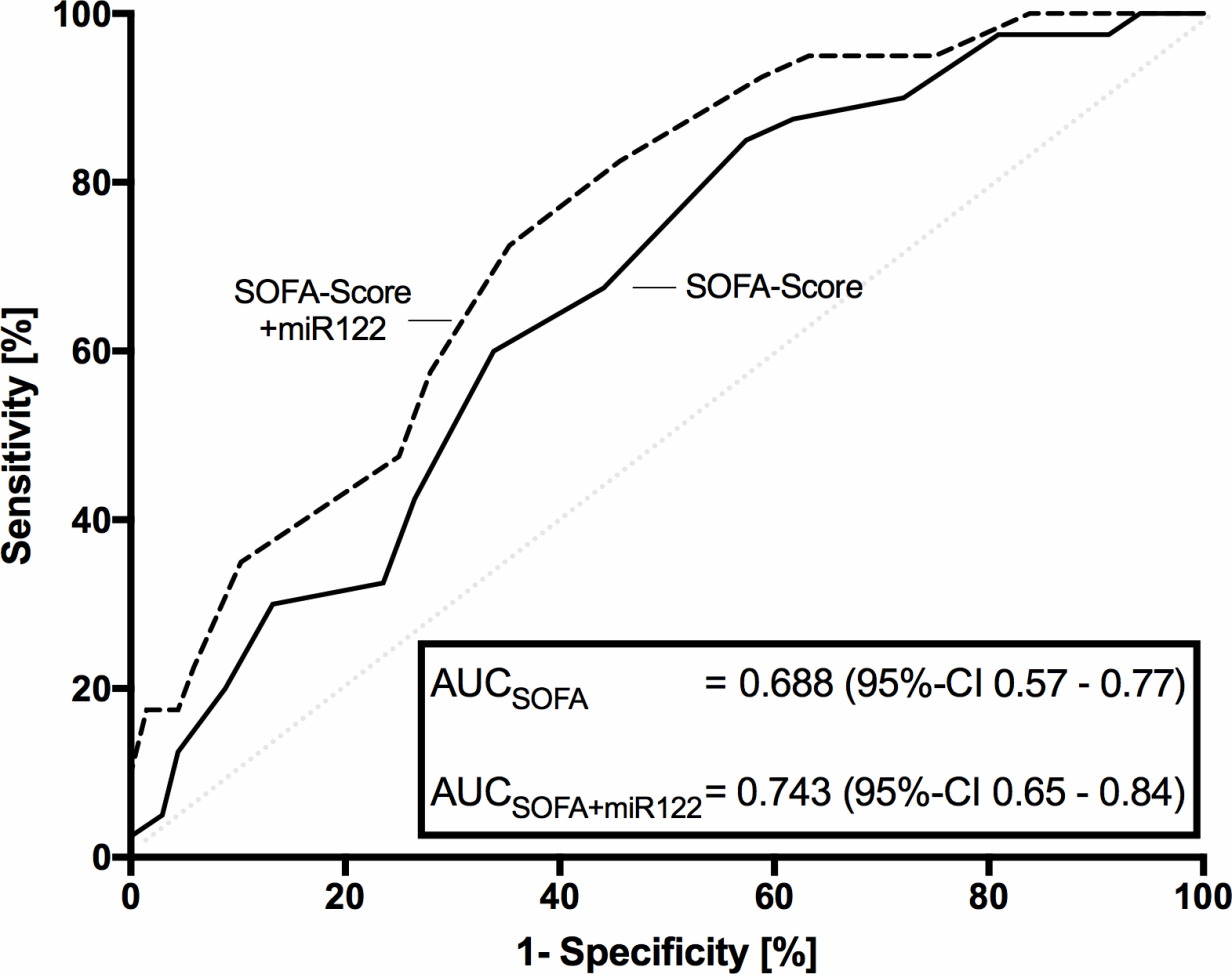
Receiver operating characteristics of SOFA-score and SOFA-score with added miR-122 expression on day 1 in patients with sepsis in relation to 30-day-mortality. Measurements on day 1 in patients with sepsis.

Finally, further ROC-curve analysis revealed, that prediction of 30-day mortality was improved markedly by addition of miR-122 to the SOFA-score (AUC 0.743, 95%-CI 0.65–0.84, Fig 4) compared to SOFA-score alone (AUC: 0.668; 95%-CI 0.57–0.77, Fig 4), without reaching significance according DeLongs test (p = 0.281). However, a prediction improvement was confirmed in the reclassification analyses, in which the SOFA-score with added miR-122 also showed increased prognostic values (NRI 0.37, p<0.001; IDI 0.07, p = 0.007).

## Discussion

This study, to our knowledge, is the first to assess whether miR-122 serum expression is helpful to discriminate between patients suffering from sepsis according to sepsis 3 criteria and those with infection without sepsis (i.e., former sepsis 1 criteria) and whether adding miR-122 expression to the SOFA-score improves the prognostic value in septic patients. We also demonstrated that miR-122 is an independent risk factor for 30-day survival.

A new sepsis definition was proposed in 2016 focusing on the early detection of acute sepsis-related organ dysfunction [4] while the SOFA-score has a history of 20 years to describe multiple organ failures in sepsis, demonstrating a good correlation between mortality and the score-value. Nevertheless, given the profound immunologic, circulatory, and metabolic abnormalities in sepsis with consecutive multiple organ dysfunction, there seems to be room for improvement of this scoring system by incorporation of new biomarkers. Specifically, goals of new biomarkers in the setting of sepsis should be a more distinction between sepsis and infection and better prediction of patients`prognosis [27]. In fact, several recent studies have explored the diagnostic and prognostic usefulness of new biomarker combinations in septic patients [28, 29].

Considering liver dysfunction in sepsis, regularly associated with a worse outcome, standard liver tests such as transaminase activity, bilirubin concentration, or liver dependent coagulation factors are usually considered [30]. However, there is limited specificity of these traditional markers for an acute liver dysfunction or injury in sepsis [31] and, therefore, these markers should be supplemented or even replaced by newer biomarkers that may be more sensitive and specific earlier. In this context, miR-122 was first introduced as a hepatic biomarker predicting direct liver injury with consecutive liver cell damage [32]. Both in animal models and in humans, increased expression of circulating miR-122 correlated with the extent of hepatic cell death after viral, alcoholic, chemical, and drug-induced liver injury [33]. Wang et al. first described a potential role of circulating miR-122 in sepsis in a cohort of 214 patients where miR-122 was identified as part of a 6 miRNA signature that predicted patients’ short and long term survival with high accuracy [34] and miR-122 was associated with 28-day mortality in diverse ICU patients with sepsis [23]. Moreover, these authors also suggested that alterations in miR-122 expression might be used as biomarker in sepsis demonstrating its altered expression in sepsis patients compared to healthy controls [23, 34].

Our results are not only in line with these former data but extend these studies by demonstrating that miR-122 expression is a strong independent predictor of survival and that its expression in patients suffering from sepsis is significantly greater compared to patients with infections but without organ-dysfunctions, referring to the new definitions of sepsis [4].

While the underlying mechanisms and pathophysiologic alterations evoking increased miR-122-expression cannot be pinpointed by our study and these alterations remain to be elucidated at the basic research level, a few speculations can be made. Since increased miR-122 concentrations indicate a poor neurological outcome in patients resuscitated from cardiac arrest, organ malperfusion may up-regulate circulating miR-122. This is supported by showing increased miR-122 expression in several ischemia and reperfusion models [22, 35]. We also could demonstrate a significant correlation of miR-122 expression with norepinephrine dosage and arterial blood pressure, suggesting that decreased organ perfusion may evoke miR-122 expression. Referring to inflammation as a trigger of miR-122 expression in a rodent-model of indirect liver injury, miR-122 increased following the appearance of increased plasma cytokine concentrations suggesting that cytokines may have induced increased miR-122 expression and/or its appearance in the blood stream [22]. In our study, there was no correlation between miR-122 expression and markers for inflammation, and miR-122-concentration was also independent of inflammation markers in a cohort of critically ill ICU patients [20]. Increased miR-122 expression was also found in patients with a prolonged activated partial thromboplastin time and low fibrinogen/antithrombin III ratio as a surrogate for aberrant liver function [21]. This is in line with our results with a significant correlation between miR-122 and INR.

In mice with sepsis, miR-122 correlates with the presence of an ongoing liver damage (according to ALT and AST serum activities) as well as hepatic cell death [20]. This also applies to critically ill patients including those with sepsis [20]. These results lead those authors to suggest, that miR-122 expression solely represents an independent and potent marker of ongoing acute liver injury and hepatic cell death. These results are comparable with our results by demonstrating a significant correlation between miR-122, AST, and ALT. Nevertheless, other findings on acute liver injury showed only a low grade cellular damage in sepsis [31], suggesting that the release of miR-122 also occurs independently of a substantial liver cell death. Accordingly, this field is not yet clarified and miR-122 is not free of significant limitations, as recently reviewed [14].

The definition of sepsis has changed markedly and our work is the first to examine miR-122 in the context of the new sepsis definition. Reference to the old SIRS-related definition of sepsis, focusing on inflammation rather than organ-dysfunction, may have blurred a useful interpretation of miR-122 as a biomarker. With the new sepsis-definition focusing on organ dysfunction impact of miR-122 might be unmasked, as supported by our present study.

### Limitations

Despite all our sepsis patients having been treated with a rather standardized multimodal regimen and although patients with chronic liver disease were excluded, undetected confounders may have distorted the results. Furthermore, although samples were stored at -80°C, we cannot entirely exclude an influence of different durations of probe storage. While repeated measurements during the further course of sepsis and tissue examinations of non-survivors may have expanded our insight associations between miR-122 expression and prognosis are limited to day 1 predictions. Nevertheless, our study population was not small and multivariate Cox regression analyses revealed miR-122 expression as an important and strong independent prognostic factor for 30-day survival in sepsis. Finally, it is unclear how miR expression measurements should be normalized to account for interindividual or intergroup variability. In our study, we used the broadly accepted spiked-in technique of non-human RNAs before miR extraction as an internal control. In this context, we demonstrated that cel-miR-54 did not differ between controls, sepsis survivors, and sepsis non-survivors (p = 0.599, S1 Fig), suggesting that cel-miR-54 can be used as a stable reference. Nevertheless, no universally accepted standard for miR measurements has been defined so far [14], limiting comparability of different studies.

## Conclusions

Increased miR-122 expression supports the discrimination between infection and sepsis, is an independent early risk factor for 30-day mortality, and improves the prognostic value of the SOFA-Score.

## Supporting information

S1 Fig Cycle Threshold [CT] of cel-miR-54 expression in control patients, sepsis survivors, and sepsis non-survivors. Cel-miR-54 levels are presented in scatter plots. Data are shown as median with interquartile range.
(PDF)

S1 File Raw data set. (XLSX)

S1 Table Baseline characteristics of the control group (infection without sepsis) and patients with sepsis according sepsis-3 criteria. (DOCX)
