# Supplementary material for: Increased circulating microRNA-122 is a biomarker for discrimination and risk stratification in patients defined by sepsis-3 criteria
Source: PLoS One. 2018 May 21;13(5):e0197637. doi: 10.1371/journal.pone.0197637 (PMC5962092; doi:10.1371/journal.pone.0197637)

S1 Fig: Cycle Threshold [CT] of cel-miR-54 expression in control patients, sepsis survivors, and sepsis non-survivors. Cel-miR-54 levels are presented in scatter plots. Data are shown as median with interquartile range.

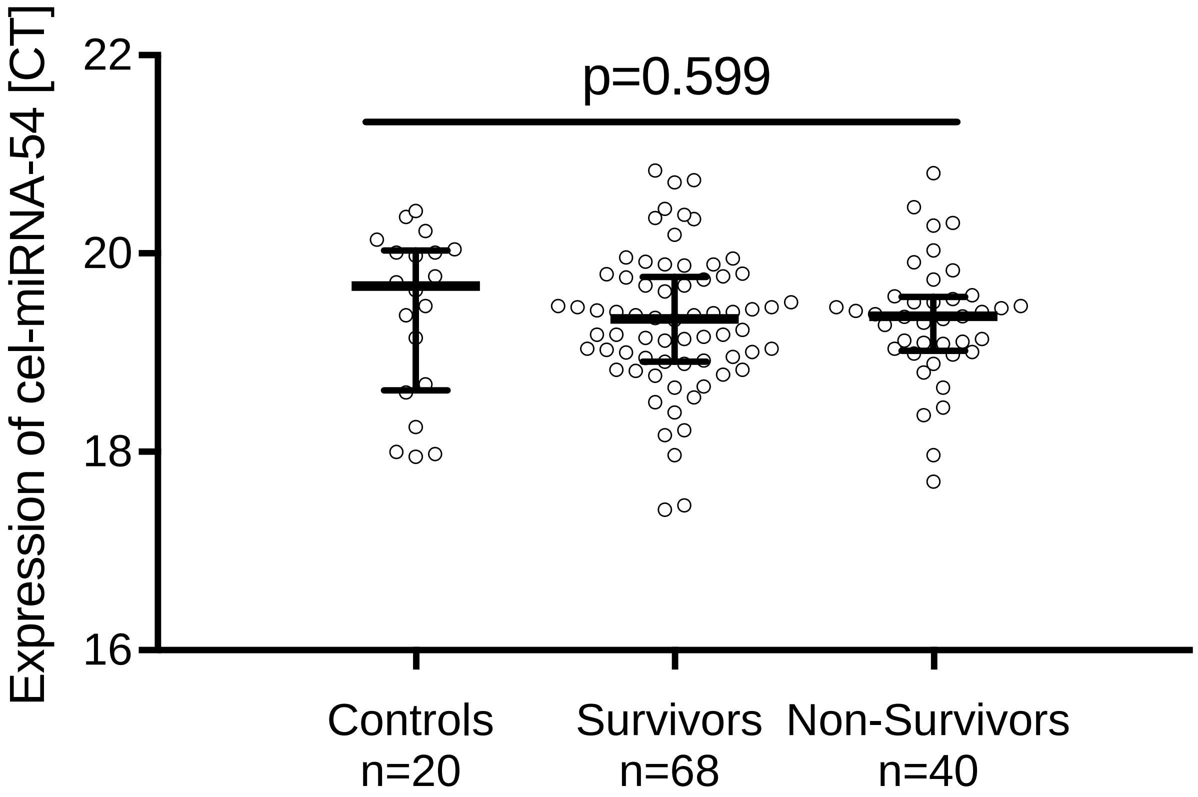

Supplement: S1 Fig — Cel-miR-54 levels are presented in scatter plots. Data are shown as median with interquartile range. (PDF) [file pone.0197637.s001.pdf]
