# Supplementary material for: Increased circulating microRNA-122 is a biomarker for discrimination and risk stratification in patients defined by sepsis-3 criteria
Source: PLoS One. 2018 May 21;13(5):e0197637. doi: 10.1371/journal.pone.0197637 (PMC5962092; doi:10.1371/journal.pone.0197637)
Supplement: S1 Table — (DOCX) [file pone.0197637.s003.docx]

**Supplement 1 Table: Baseline characteristics of the control group (infection without sepsis) and patients with sepsis according sepsis-3 criteria**

| **Variable** | **Controls**  **n=20** | **Sepsis**  **n=108** | **P-value** |
| --- | --- | --- | --- |
| Age *yrs.* (range/± SD) | 52.3 (24-82/±17.2) | 55.6 (18-86/±15.7) | 0.395 |
| Male gender (%) | 9 (45) | 64 (59) | 0.267 |
| Body mass index (*kg/m^2^*) | 26.5 (±5.4) | 27.1 (± 5.8) | 0.634 |
| Medical history, no. (%) |  |  | 0.894 |
| - Cardiovascular disease | 6 (30%) | 25 (23%) |  |
| - Gastrointestinal disease | 4 (20%) | 31 (29%) |  |
| - Gastrointestinal cancer | 1 (5%) | 6 (5%) |  |
| - Hematooncological disease | 0 (0%) | 4 (4%) |  |
| - Lung disease | 7 (35%) | 23 (21%) |  |
| - Skin and soft tissue infection | 0 (0%) | 4 (4%) |  |
| - Trauma | 1 (5%) | 3 (3%) |  |
| - Urogenital disease | 1 (5%) | 8 (7%) |  |
| - Other | 0 (0%) | 4 (4%) |  |
| C-reactive protein concentration (*mg/dl)* | 11.5 [5.8-18.0] | 12.1 [6.0-19.7] | 0.948 |
| Procalcitonin concentration (*ng/m*l) | 1.6 [0.9-3.1] | 3.3 [1.1-12.6] | 0.019 |
| Interleukin-6 concentration (*pg/ml*) | 99.8 [21.3-248.9] | 93.8 [40.4-460.0] | 0.651 |
| Leukocyte concentration (**10^9^/l*) | 12.4 [9.6-19.6] | 14.0 [9.2-20.3] | 0.832 |
| AST activity (*U/l)* | 62.0 [51.3-89.8] | 59.0 [28.5-171.5] | 0.678 |
| ALT activity (*U/l)* | 44.5 [31.8-66.3] | 44 [22.5-157.0] | 0.975 |
| Total bilirubin concentration (*mg/dl)* | 0.5 [0.3-0.7] | 1.4 [0.6-2.9] | <0.001 |
| LDH activity (*U/l*) | 286 [201.5-383.5] | 385 [252-563] | 0.091 |
| INR | 1.2 [1.0-1.4] | 1.3 [1.1-1.5] | 0.170 |
| Platelet concentration *(/nl)* | 204 [167-229] | 108 [56.5–198] | 0.001 |
| Horowitz-index (P_a_O_2_/FiO_2_), (mmHg) | 412.5 [356-487] | 313 [205-379] | <0.001 |
| SAPS II | 22.65 (±7.4) | 46.4 (±17.5) | <0.001 |
| Gram positive isolates only (%) | 7 (45%) | 43 (40%) | 0.876 |
| Gram negative isolates only (%) | 6 (30%) | 35 (32%) |  |
| Mixed bacterial isolates (%) | 3 (15%) | 14 (13%) |  |
| Viral isolates (%) | 0 (-) | 0 (-) |  |
| Fungal isolates (%) | 0 (-) | 2 (2%) |  |
| Negative cultures (%) | 4 (20%) | 14 (13%) |  |

Data are presented as n (%); means (± SD), medians (25th, 75th percentile), AST: Aspartate aminotransferase ALT: Alanine aminotransferase, LDH: Lactate dehydrogenase, INR: International Normalized Ratio; SAPS II: Simplified Acute Physiology Score.
